# Genome Sequences of Measles Virus D4 and D8 Genotypes from India

**DOI:** 10.1128/MRA.00538-21

**Published:** 2021-07-15

**Authors:** Divya R. Bhattad, Madhukar B. Kamble, Sunil R. Vaidya

**Affiliations:** a Virus Registry and Virus Repository Group, ICMR National Institute of Virology, Pune, India; Queens College CUNY

## Abstract

The genomes of 15 measles viruses isolated in 2006 to 2017 from patients <16 years of age with fever and skin rashes from four states and two union territories of India were sequenced. Study genomes were phylogenetically analyzed using 143 Indian and global genomes. The study reconfirms two lineages of D4 isolates and three lineages of D8 isolates from India.

## ANNOUNCEMENT

Measles virus (MeV) (genus *Morbillivirus*, family *Pramyxoviridae*) is a close relative of a rinderpest virus that was described as a pathogen of cattle (1). Molecular clock analysis showed the origin of measles in the sixth century (2). A full-genome sequence of wild-type MeV was reported in 2000 (3), and subsequently the number increased. A 15,894-nucleotide-long MeV RNA genome contains six structural genes (nucleocapsid [N], phosphoprotein [P], matrix [M], fusion [F], hemagglutinin [H], and large protein [L]) and two nonstructural genes (V and C) (GenBank accession numbers AF128253.1 and NC_001498.1).

The molecular epidemiology of MeVs in India from 2005 to 2015 was studied (4, 5) and indicated major circulation of MeV D4 and D8 genotypes and limited transmission of B3 and D7 genotypes. The use of a matrix-fusion (M-F) intergenic/noncoding region was documented with the current N/H gene-based genotyping approach (6). Previously, 43 Indian MeVs were grouped into two lineages of D4 genotypes and three lineages of D8 genotypes (7). The spatiotemporal transmission dynamics of MeV D genotypes indicated introduction of D4 and D8 into India in 1991 and 1994, respectively (8).

In this work, 3 MeV D4 isolates and 12 MeV D8 isolates obtained from four states and two union territories of India were sequenced (Table 1). These MeVs were isolated from 2006 to 2017 from patients <16 years of age with suspected measles, using either throat swab or urine samples.

**TABLE 1:** Details of Indian MeV isolates (*n* = 15)

MeV isolate no.	Isolation source	MeV isolate name	GenBank accession no.	Yr of isolation	Location of isolation
1	Throat swab	MVi/Pune.IND/10.13/1_D8	MW916919.1	2013	Pune, Maharashtra
2	Urine	MVi/Jamnagar.IND/23.15_D8	MW916920.1	2015	Jamnagar, Gujarat
3	Throat swab	MVi/Sundargarh.IND/10.14_D8	MW916921.1	2014	Sambhalpur, Odisha
4	Throat swab	MVi/Haridwar.IND/06.14/3_D8	MW916922.1	2014	Haridwar, Uttarakhand
5	Urine	MVi/Daman.IND/7.15_D8	MW916923.1	2015	Daman and Diu
6	Throat swab	MVi/Dadra&NagarHaveli.IND/18.15_D8	MW916924.1	2015	Silvassa, Dadra, and Nagar Haveli
7	Urine	MVi/Jamnagar.IND/18.15_D8	MW916925.1	2015	Jamnagar, Gujarat
8	Urine	MVi/Jamnagar.IND/18.15/2_D8	MW916926.1	2015	Jamnagar, Gujarat
9	Throat swab	MVi/Anand.IND/33.15/2_D8	MW916927.1	2015	Anand, Gujarat
10	Throat swab	MVi/Pune.IND/16.14_D4	MW916928.1	2014	Pune, Maharashtra
11	Urine	MVi/Jamnagar.IND/4.17/1_D4	MW916929.1	2017	Jamnagar, Gujarat
12	Throat swab	MVi/Krishnagiri.Ind/12.06_D8	MW916930.1	2006	Krishnagiri, Tamil Nadu
13	Throat swab	MVi/Villupuram.Ind/13.06_D8	MW916931.1	2006	Villupuram, Tamil Nadu
14	Throat swab	MVi/Villupuram.Ind/03.07_D8	MW916932.1	2007	Villupuram, Tamil Nadu
15	Urine	MVi/Perambalur.Ind/17.07_D4	MW916933.1	2007	Perambalur, Tamil Nadu

All isolates were grown in Vero/hSLAM cells (5). RNA was extracted from 0.5 ml of tissue culture fluid with the QIAamp viral RNA kit (Qiagen). Reverse transcription (RT)-PCR of different genes was performed using 25 primer pairs (overlapping regions) and a one-step RT-PCR kit (Invitrogen), and purified amplicons (ExoSAP-IT; Affymetrix Inc.) were sequenced by Sanger’s method using the BigDye Terminator v3.1 cycle sequencing kit (Thermo Fisher Scientific) on a DNA Analyzer (ABI 3730XL) (7). Fifty-eight Indian and 100 global MeV genomes were utilized for the phylogenetic analysis. Genomes were aligned using the MAFFT tool, the alignments were trimmed to remove leading and trailing gaps, and a phylogenetic tree was generated using the maximum likelihood method (IQ-TREE server). Default parameters were used for all software unless otherwise specified.

**FIG 1:**
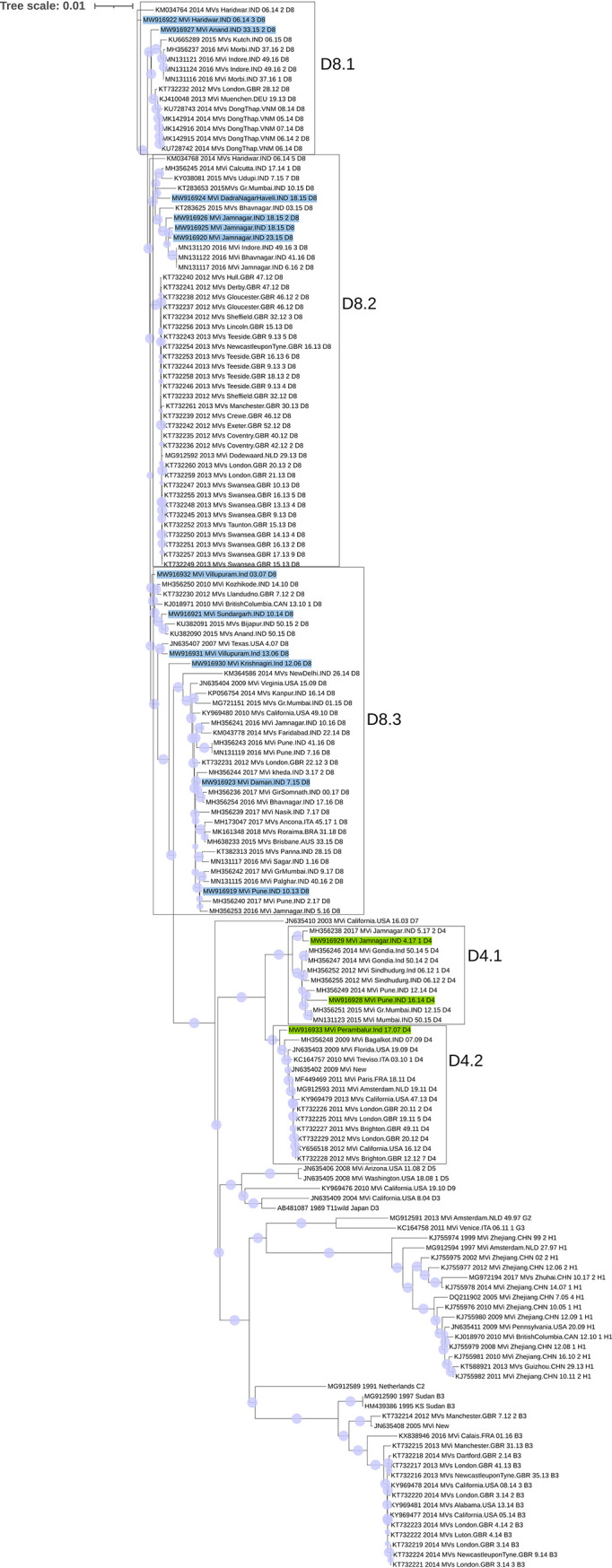
Phylogenetic tree of genomes of 15 study isolates (D4 genomes highlighted in green and D8 genomes in blue) along with 143 Indian and global isolates. The GTR+I+Gamma model was used for the phylogeny. One thousand bootstrap replicates were used, and gray circles indicate bootstrap support of >70%.

MeV isolates revealed standard lengths except for 2 D4 isolates (15,900 nucleotides), with a GC content of 47%. Of the 3 D4 isolates, the Pune-2014 and Jamnagar-2014 isolates clustered in the D4.1 sublineage and the Perambalur-2007 isolate in the D4.2 sublineage (7). All D8 isolates were subclustered in D8.1 and D8.2 (7). Interestingly, the Krishanagiri-2006 and Villupuram-2006 isolates were found to join as the outermost node of the D8 lineage (Fig. 1). Mutations K387E (in the hemagglutinin noose epitope) and S318R (in the loop epitope) of the H protein were observed in Villupuram-2006, and I473K was observed in Krishanagiri-2006. An insert of CCCCCCC in the M gene and deletion of 1 nucleotide in the F gene were evident in 2 D4 isolates. Previously, D4 strains from other countries including India reported such insertions (6, 7). The nucleotide divergence was found to be higher for M-F genes (D4, 6.2%; D8, 3%) than for H (D4, 1.5%; D8, 1%) and N (D4, 1.4%; D8, 1.8%) genes.

Previously, WHO-SEAR and the government of India agreed to eliminate measles and control rubella by 2020 (9); now, this goal has been modified to eliminate both by 2023 (10). Thus, use of genome sequencing is crucial to track the transmission of MeVs during elimination programs.

### Data availability.

Genome sequences are available in GenBank under accession numbers MW916919.1 to MW916933.1 (Table 1).
